# An adaptive rejection sampler for sampling from the Wiener diffusion model

**DOI:** 10.3758/s13428-022-01870-z

**Published:** 2022-10-19

**Authors:** Raphael Hartmann, Constantin G. Meyer-Grant, Karl Christoph Klauer

**Affiliations:** 1 Department of Psychology, University of Marburg, Gutenbergstrasse 18, D-35032 Marburg, Germany; 2 Department of Psychology, University of Freiburg, Engelbergerstrasse 41, D-79085 Freiburg im Breisgau, Germany

**Keywords:** Sampling methods, Adaptive rejection sampling, Wiener diffusion model

## Abstract

The Wiener diffusion model with two absorbing boundaries is one of the most frequently applied models for jointly modeling responses and response latencies in psychological research. We consider four methods for sampling from the model with and without variability in drift rate, starting point, and non-decision time: Inverse transform sampling, rejection sampling, and two new methods based on adaptive rejection sampling (ARS). We implement these four methods in an *R* package, validate the methods, and compare their sampling speed in different settings. All four implemented methods provide samples that follow the intended distributions. The ARS-based methods, however, outperform the other methods in sampling speed as the requested sample size increases. We provide guidelines for when using ARS is more efficient than using traditional methods and vice versa.

Binary choice tasks, in which a rapid decision between two alternatives is required, are an indispensable component in the toolbox of many experimental psychologists. In studying response behavior in such tasks, researchers are interested in making inferences as to the cognitive mechanisms underlying the observed decisions. For this purpose, the application of cognitive modeling is often helpful.

The *Wiener diffusion model* (WDM; Ratcliff, 1978) is arguably one of the most prominent cognitive models of such tasks (for extensive reviews see Ratcliff & McKoon, 2008; Ratcliff et al., 2016). It assumes that in order to make a binary choice, information on the current stimulus display is sampled over time. Such information can—dependent on the task at hand—constitute evidence in favor of one decision and, by implication, against the other. Entering a mental decision-state is tantamount to accumulating a critical amount of evidence in favor of one of the two response options.

At its core, the WDM is based on a two-dimensional *Wiener process* with *drift rate* $\nu$, variance $\sigma = 1$ (without loss of generality), and two *absorbing boundaries* at 0 and *a*, where $a > 0$. The value $X(t) \in \mathbb {R}$ that the process takes at any given time *t* ≥ 0 corresponds to the level of evidence accumulated thus far and needs to be interpreted relative to the so-called *starting point z* := $X(0), \mathrm{where}\; 0 < z < a$. This latter quantity models the initial state of evidence before any stimulus-related information has been accumulated. The boundaries of the process are absorbing in so far as the process remains stationary as soon as *X*(*t*) = 0 or *X*(*t*) = *a* and the decision itself is determined by the boundary at which the absorption occurs. These boundaries hence correspond to the critical evidence for the respective associated decision and the time required to reach one of the boundaries—referred to as *first-passage time*—equals the time required for the decision process to complete.

The tendency of the process to approach either the upper or the lower boundary is determined by the sign of the drift rate $\nu$. That is, if $\nu$ is negative (positive) the process will tend to the lower (upper) boundary. The strength of this tendency is represented by the drift rates’ absolute magnitude. This parameter is generally assumed to reflect the efficiency of information uptake.

Within the WDM, speed–accuracy trade-off occurs because erroneous decisions become less likely and decision times longer as the *boundary separation* increases (i.e., the greater *a*). Moreover, the initial evidence state can be expressed as the relative position *w* of the starting point between the upper and lower boundary such that a value of *w* = 0.5 corresponding to the starting point *z* = *a*/2 (i.e., both boundaries having the same distance to the starting point *z*). This parameter (*w*) can accommodate possible response biases: The closer the process starts to one boundary (relative to the other), the greater is the probability of absorption at that boundary for a given drift rate $\nu$.

Response times not only comprise the decision time itself but also other components unrelated to the decision process, such as stimulus encoding and motor execution, and psychological models like the WDM additionally propose an additive *non-decision time* *t*_0_ to capture such components. Another important extension of the WDM is the introduction of within-person (i.e., between-trial) variability in the drift rate $\nu$, the relative starting point *w*, and the non-decision time *t*_0_ (see, e.g., Ratcliff & Smith, 2004); that is, these quantities are assumed to be random variables themselves. A common assumption about the distributions for these random variables are: $\nu$ follows a normal distribution with mean $\mu_\nu$ and standard deviation $s_\nu$, *w *follows a uniform distribution with minimum and maximum equal $\mu_{w}-s_{w}/2$ and $\mu_{w}+s_{w}/2$, respectively, and *t*_0_ follows a uniform distribution with minimum and maximum equal $min_{t_{0}}$ and $min_{t_{0}}$ + $s_{t_{0}}$, respectively (see, e.g., Hartmann & Klauer, 2021).

The WDM possesses many desirable properties, which are pivotal for the model’s success. First, the WDM has received ample empirical support in diverse psychological domains (e.g., Arnold et al., 2015; Lerche & Voss, 2019; Ratcliff et al., 2016; Voss et al., 2004). Second, as argued by these authors, when the model fits the data, it provides a consistent account of the data in terms of psychologically interpretable processes. Notably, a process of evidence accumulation, associated with the decision time, is separated from encoding and motor processes, associated with the non-decision time. Third, it makes full use of both accuracy and latency data and can account for both data types simultaneously (see, e.g., Ratcliff, 1978). Lastly, the WDM is able to model speed–accuracy trade-off as well as response bias in a natural fashion. As pointed out by Jones and Dzhafarov (2014), the account provided by the WDM hinges, however, on specific distributional and structural auxiliary assumptions, and alternative accounts of a given dataset implying different psychological interpretations can always be constructed based on different sets of auxiliary assumptions. For such reasons, it is desirable to validate the interpretation of the WDM parameters in terms of psychological processes via selective-influence studies in each domain of application as elaborated on by Klauer (2014).

However, the WDM also possesses a particular technical disadvantage, which relates to its first-passage time distribution. This distribution is of paramount interest for practical applications, since it contains all relevant information for modeling the decision process (i.e., the decision time and the decision itself). Technically, the probability density function (PDF) of the first-passage time distribution can be represented by two separate functions—each proportional to the PDF conditioned on absorption at the lower (upper) boundary. Note that each of those separate functions does not integrate to one, but instead to the probability of absorption at the respective response boundary. However, the PDF as well as the cumulative distribution function (CDF) of the first-passage time distribution are not available in closed form. Instead, the PDF and CDF for the first-passage time distribution of a Wiener process with two absorbing boundaries are expressed in terms of an infinite series (see, e.g., Cox & Miller, 1965, Chapter 5.7). Although many advances regarding the applicability of the WDM have been made in recent years (see e.g., Blurton et al., 2012; Blurton et al., 2017; Gondan et al., 2014; Navarro & Fuss, 2009) this fact still hinders many practical applications.

One of the immediate consequences of this problem is that all sampling algorithms available to date involve computationally expensive numerical procedures, which render computation times infeasible for many sampling-heavy applications. Tuerlinckx et al. (2001), for example, provide an overview of four approaches to sample first-passage times from WDMs and in a more recent paper Drugowitsch (2016) introduces a rejection sampling (RS) method that rapidly samples first-passage times when the relative starting point is 0.5. Nevertheless, the sampling time of all these methods increases drastically with increasing sample size. We therefore derived a new sampling approach building on the works of Gilks and Wild (1992) who suggest an adaptive rejection sampling (ARS) procedure for Gibbs sampling, which needs fewer and fewer rejection steps the more samples are drawn and therefore keeps the sampling time relatively low. In addition, we developed a variation of the ARS method that deals with some of the ARS’s weaknesses. More details about the ARS method and its variation are presented in the next section, where we also present two existing methods with which we compare the new methods. In short, the ARS method outperforms the other methods in cases with no within-person variability, with within-person variability only for the relative starting point, or in many cases with sample sizes larger than 100,000. We find that it is up to five times faster than the other methods in these cases.

## Methods

### Adaptive rejection sampling

ARS (Gilks & Wild, 1992) is a RS method. It requires an *envelope* function $g_{u}(x)$ and a *squeezing* function $g_{l} (x)$ such that $g_{l} (x) \leq g(x) \leq g_{u}(x)$ for all *x*, where $f(x)=Cg(x)$ is the PDF from which one wishes to sample and *C* is a constant. The envelope function should be proportional to a PDF from which it is easy to sample. Sampling from $f(x)$ proceeds by taking a proposal sample *x*^∗^ from $g_{u}(x)$ and sampling a value *u* from a uniform distribution. If $u\leq g_{l}(x^{*})/g_{u}(x^{*}), x^{*}$ is accepted, obviating the costly evaluation of $g(x^{*})$. If the inequality does not hold, $g(x^{*})$ is evaluated and it is checked whether $u \leq g(x^{*})/g_{u}(x^{*})$. If this inequality holds, $x^{*}$ is accepted and otherwise rejected. The new idea of ARS is to use an envelope and a squeezing function which are updated after each rejection step.

**Fig. 1:**
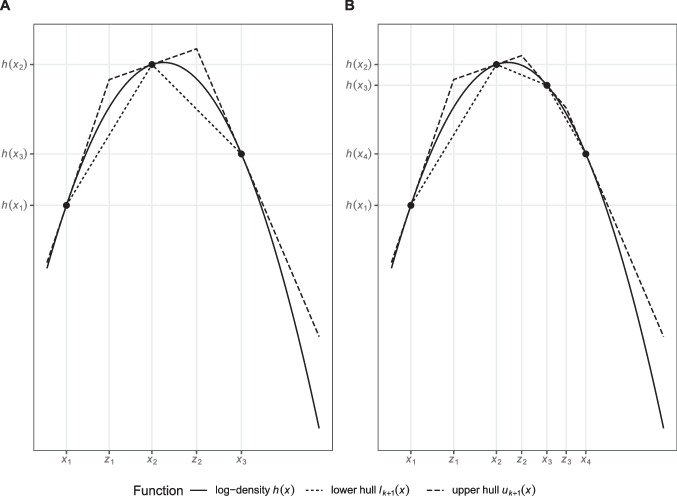
Exemplary log-density with lower and upper hull function. *Note.* The log-density *h*(*x*) denotes the log-transformed PDF of an exemplary distribution with a log-concave PDF. Panel **A** depicts the log-density along with its lower and upper hull, *l*_*k*_(*x*) and *u*_*k*_(*x*), respectively, for *k* = 3 points and $T_{k}= T_{3}= \{x_{1}, x_{2}, x_{3}\}$. Panel **B** depicts the log-density along with its lower and upper hull for *k* + 1 = 4 points and $T_{k+1}= T_{4}= \{x_{1}, x_{2}, x_{3}, x_{4}\}$. Here a proposal value *x*^∗^ was added to *T*_*k*_ (becoming *x*_3_ in the set $T_{k+1}$), and the lower and upper hull were recalculated, resulting in $l_{k+1}(x)$ and $u_{k+1}(x)$, respectively. The *z*_*i*_’s denote the intersection points of the piecewise linear upper hull functions in both panels

As mentioned by Gilks and Wild (1992), there are some prerequisites that must be fulfilled in order for ARS to yield valid samples; the function *g*(*x*) must be (a) differentiable on the whole domain of *x* and (b) log-concave, that is, $h(x):=\log (g(x))$ must be concave. The envelope function is constructed by (1) taking some initial values $T_{k}=\{x_{i}: x_{i} \leq x_{j}\; \mathrm{for}\; i < j\; \mathrm{and} \; i, \, j\, \in \{1,..., k\}\}$, (2) calculating the linear tangents of *h*(*x*) at these points, (3) calculating the *x*-coordinates of the points, *z*_*i*_, at which adjacent tangents intersect, and (4) constructing an *upper hull* of *h*(*x*), *u*_*k*_(*x*), as a piecewise linear function connecting these points. This provides an envelope function, $\exp (u_{k}(x))$, from which it is easy to sample. The *lower hull* *l*_*k*_(*x*) is constructed as a piecewise linear function connecting the points (*x*_*i*_, *h*(*x*_*i*_)) for adjacent *x*_*i*_’s. This provides a squeezing function, $\exp (l_{k}(x))$. Panel A of Fig. 1 shows an exemplary log-density function *h*(*x*) along with its upper hull function *u*_*k*_(*x*) and lower hull function *l*_*k*_(*x*) for *k* = 3.

A proposal value *x*^∗^ is sampled first: Let *J*_*i*_ be the integral for the piecewise function $\exp (u_{k}(x))$ from *z*_*i*− 1_ to *z*_*i*_, *J*_0_ = 0, and $J = {\sum }_{j=0}^{k} J_{j}$. First, *u*_prop_ is uniformly sampled from $[0, 1]$, then we find $i \in \{1,...,\,k\}$, such that $({\sum }_{j=0}^{i-1} J_{j})/J \leq u_{\text {prop}} < ({\sum }_{j=0}^{i} J_{j})/J$, and then we have to invert for

1 $$u_{\text{prop}}^{*} = \frac{u_{prop} - ({\sum}_{j=0}^{i-1} J_{j})/J}{J_{i}}$$

by calculating *x*^∗^, such that $u_{\text {prop}}^{*} = {\int \limits }_{z_{i-1}}^{x^{*}} \exp (u_{k}(x)) dx ~ /~ J_{i}$ to get the proposal value. Once the proposal value *x*^∗^ is sampled, the same rejection step as in RS is used: If for a uniformly sampled value *u* (from [0, 1]) either

2 $$u \leq \exp(l_{k}(x^{*}) - u_{k}(x^{*}))$$

or

3 $$u \leq \exp(h(x^{*}) - u_{k}(x^{*}))$$

holds, the value will be accepted and rejected otherwise. If *h*(*x*^∗^) needs to be calculated during this rejection step (i.e., the inequality in Eq. 2 does not hold), an *updating step* will be conducted by including *x*^∗^ in *T*_*k*_ resulting in *T*_*k*+ 1_ and by recalculating the upper and lower hull for *T*_*k*+ 1_, which results in *u*_*k*+ 1_(*x*) and *l*_*k*+ 1_(*x*), respectively. Panel B of Fig. 1 shows how such a proposal value *x*^∗^ is added to *T*_*k*_ to form *T*_*k*+ 1_ and how the new upper and lower hull functions approximate the log-density function even better (for more technical details, see Gilks & Wild, 1992). The advantage of the updating step is that the envelope and squeezing function will thereby come to approximate *g*(*x*) increasingly closely with every new updating step which will make it increasingly more likely that values sampled from the envelope function can be accepted without the costly evaluation of *g*(*x*).

The conditional first-passage time PDF of the WDM given absorption at the lower (upper) boundary is not log-concave. Therefore, we decided to use *change-of-variables* with the following transformation of **T**, the first-passage time, to get a new variable **A**:

4 $$\mathbf{A} = {v}(\mathbf{T}) = \frac{\log(\mathbf{T})-\alpha_{0}}{s_{\alpha}},$$

where *α*_0_ is a location parameter and *s*_*α*_ a scaling parameter.^1^ The resulting PDF of **A** is then

5 $$\begin{array}{@{}rcl@{}} f_{A}(\alpha) &=& \left\lvert \frac{\partial}{\partial \alpha} {v}^{-1}(\alpha)\right\rvert \times f_{T}({v}^{-1}(\alpha))\\ &=& s_{\alpha} \exp(s_{\alpha} \alpha + \alpha_{0}) \times f_{T}(\exp(s_{\alpha} \alpha + \alpha_{0})). \end{array}$$

However, this PDF, like the original first-passage time PDF, can only be represented as an infinite series. This fact renders rigorously proving log-concavity difficult and we were unfortunately not able to devise a way to do so. Nevertheless, a number of arguments make us confident that this new PDF (*f*_*A*_(*α*)) is log-concave. First, using the same change-of-variables as in Eq. 4 for the PDF of the first-passage time of a Wiener process with only one absorbing boundary (i.e., the inverse Gaussian PDF) results in a function for which log-concavity can in fact be proven (see Appendix “A”). Second, the second partial derivative of the new PDF with respect to *α* can be calculated numerically, and in an extensive simulation study described in the Section “Log-concavity for ARS”, no violations of log-concavity were found. Third, the above-described ARS algorithm would crash in the updating step if updating entered a region of *g*(*x*) for which log-concavity is violated; but the new sampling method performs very stably across many millions of sampling and updating steps. Fourth, the validity of the sampled values can be checked in terms of whether they follow the intended first-passage time distribution, and as described below, they did so in extensive simulations (see Section “Accuracy of the sampling methods”). Nevertheless, we acknowledge that the absence of a formal proof of log-concavity is a weakness of the proposed method.

Use of the ARS method does not only require the PDF, but also its derivative with respect to *α*. In this respect, the new method builds on Hartmann and Klauer (2021) who provided an efficient algorithm for computing this derivative.

Assuming that the PDF of random variable **A** is log-concave, one can sample *α* values from this new distribution using ARS. In order to get first-passage times, one can back-transform the sampled *α* values using *v*^− 1^(*α*) to get random samples from the first-passage time distribution of the WDM. Note that the ARS method is a method to sample from the conditional first-passage time PDF given absorption at the lower (upper) boundary. When sampling both the absorbing boundary and first-passage time, the boundary is first sampled from a simple Bernoulli distribution with the appropriate absorption probabilities

6 $$\begin{array}{@{}rcl@{}} \mathrm{P}(\text{absorption at upper bound}) &=& \frac{1-\exp(2\nu aw)}{\exp(-2\nu a(1-w))-\exp(2\nu aw)}\\ \mathrm{P}(\text{absorption at lower bound}) &=& 1-\mathrm{P}(\text{absorption at upper bound}) \end{array}$$

followed by sampling the first-passage time from the distribution of first-passage times conditional on the sampled boundary.^2^

### Alternative sampling methods

In the following three paragraphs, we describe three sampling methods with which we compared the ARS. We restrict ourselves to sampling methods that sample from the exact first-passage time distributions (up to a prespecified precision for numerical errors) rather than from approximations thereof. Diffusion processes can be approximated in terms of a random walk with small time steps (e.g., Diederich & Busemeyer, 2003) or in terms of discrete approximations of the stochastical differential equation describing the diffusion process (e.g., Smith, 2000), and sampling methods have been proposed that sample from such approximations (Tuerlinckx et al., 2001). Tuerlinckx and colleagues also proposed an exact RS method that we do not consider, because a later proposal by Drugowitsch (2016), which we do consider, improves on it. All of the sampling methods that we consider can be modified to sample from a first-passage time distribution with truncation from above. We initially describe how these algorithms generate samples from a Wiener first-passage time distribution and will later delineate how within-person variability can be incorporated.

#### Inverse transform sampling (ITS)

ITS is a widely applicable method to generate random samples. In the present case, we start from the CDF *F* of first-passage times given absorption at the lower (upper) boundary and use its inverse function, *F*^− 1^ (i.e., the quantile function), to transform random draws from a uniform distribution into samples from the target distribution. More precisely, if $\mathbf {U} \sim \mathcal {U}(0,1)$ (i.e., **U** is uniformly distributed on [0, 1]) then *F*^− 1^(**U**) will be distributed as first-passage time of the WDM given absorption at the lower (upper) boundary (Devroye, 1986). In the case of the Wiener first-passage time distribution, there unfortunately exists no analytic quantile function. In order to apply ITS, we therefore have to employ a numeric root finding algorithm. We used a simple, yet efficient bisection method for this purpose. Like ARS, ITS as stated is a method to sample from the conditional first-passage time PDF given absorption at the lower (upper) boundary.

#### Rejection sampling (RS)

As already mentioned, Drugowitsch (2016) proposed an RS algorithm that is quite fast when the relative starting point is set to 0.5, although as discussed by Drugowitsch (2016), it can be extended to cases with relative starting points other than 0.5, which we did. An RS algorithm uses envelope functions—or rather proposal distributions—from which it is easy to sample. These proposal distributions when multiplied with a suitable factor need to dominate the distribution of interest. The closer the proposal resembles the distribution of interest (i.e., their function values are close to each other) the fewer rejections are needed. Drugowitsch uses different proposal distributions based on the exponential distribution, a scaled-inverse *χ*^2^-distribution, and the inverse Gaussian distribution (for technical details, see Drugowitsch, 2016).

#### Pseudo-adaptive rejection sampling (P-ARS)

This sampling method builds on the ARS method presented above. The difference to ARS is that after each sample the upper and lower hull are discarded in the P-ARS, that is, the hull functions are updated until the first sample is drawn, but the updates are not kept for the next sample. The cost of this is a considerable slowdown due to the calculation of the hull functions for each new sample. This is especially momentous when using no within-person variabilities. Therefore, P-ARS is predicted to be slower than ARS in these cases. However, when within-person variability is introduced, P-ARS avoids costly numerical integration, as described next, and thus might outperform ARS for this reason in these cases.

### Sampling from the WDM with within-person variability

Given within-person variability, the ARS method requires numerical integration across the parameters (drift rate and/or starting point) with trial-by-trial variability. The ITS, RS, and P-ARS methods outlined above can sidestep costly numerical integration by adopting a two-step sampling scheme: First, parameters with trial-by-trial variability are sampled; next, a boundary and first-passage time are sampled from the WDM with these parameters and no within-person variability.

Note, however, that this sampling scheme needs to be modified when sampling conditional on a specific response such as when sampling first-passage times at the lower threshold. The above two-step procedure will ensure that the respective parameters are distributed according to their assumed distributions, but the subsequently sampled first-passage times conditional on absorption at a specific boundary will *not* follow the intended distribution. This phenomenon is caused by the fact that the larger the sampled starting point and/or drift rate is, the more likely absorption at the upper boundary becomes compared to absorption at the lower boundary and vice versa. Consequently, if one only considers those processes that absorb at the upper (lower) boundary, the distribution of parameters will deviate from the unconditional distribution of parameters in so far as higher (lower) parameter values are overrepresented compared to the unconditional parameter distribution. One therefore needs to account for this fact by conducting an additional rejection step such that parameters are sampled based on their likelihood of leading to an absorption at the relevant boundary.

To obtain the desired behavior of the sampler, one therefore first samples the random parameters according to their respective (unconditional) distributions. In a second step, a randomly sampled value *u* (from $\mathcal {U}(0,1)$) is drawn and the just sampled parameters are accepted only if *u* is less than or equal to the probability of a Wiener process with the proposed parameters being absorbed at the boundary of interest. This additional step introduces an obvious computational cost.

### *R* Package

The implementations of the sampling methods introduced here build on Hartmann and Klauer’s (2021) work which derived efficient methods for computing PDF, CDF, and their partial derivatives as implemented in an *R* package called WienR (Hartmann & Klauer, 2021). We extend this package by a sampling function with which one can sample from a (truncated) first-passage time distribution (conditional on the absorption at one of the two boundaries) of the WDM with up to seven parameters.^3^

#### Installation and usage

The *R* package WienR is listed in the comprehensive *R* archive network (CRAN). Therefore, it can be installed by using the function call install.packages("WienR").

The newly implemented sampling function sampWiener()^4^ can be used like many other sampling functions in *R*. Its first argument is N, the number of samples to be drawn. The second to eight arguments are the parameters of the model a, v, w, t0, sv, sw, and st0 which refer to, in order, the upper boundary, the (mean) drift rate, the (mean) relative starting point, the (minimal) non-decision time, the within-person variability of the drift rate, the within-person variability of the relative starting point, and the within-person variability of the non-decision time. The next argument is response where "upper", "lower", or "both" boundaries can be specified. There are default values for t0, sv, sw, and st0 since these arguments are not required for the standard WDM and therefore are set to zero. The argument response has "both" as default.

In addition to those necessary parameters, there are six further parameters, starting with bound, that is, the truncation from above. Its default is set to infinity. Next is method where the four previously described sampling methods can be chosen. The options are "ars" (the default), "rs", "its", and "p-ars" which stand for the methods ARS, RS, ITS, and P-ARS, respectively. The third optional parameter is precision where one can specify with which precision the evaluations of PDF, CDF, and partial derivatives are calculated (its default is 1e-12). The fourth parameter is n.threads which is the number of threads to be used (default is one). The two last arguments are special in that they only concern the ARS method. The first of those two, ars_list, requires a list consisting of information about the upper and lower hull functions as well as some additional information. Its default is NULL, meaning no information is used. The second of those two, ARS_STORE, is used to specify whether the information about upper and lower hull etc. should be stored (default is FALSE).

The output of the function is a list containing the *N* first-passage times q and responses response as well as the whole function input as a string. In addition, if the ARS method is selected and ARS_STORE is set to TRUE, a list containing the information about upper and lower hull as well as some additional information is stored. If both response boundaries are used this list consists of two sub-lists consisting of the equivalent information for both the response "upper" and the response "lower", respectively.

Here is an example function call to sampWiener() with a sample size N of 10, an upper boundary a of 1, a drift rate v of 0.3, a relative starting point w of 0.6, a non-decision time t0 of 0.2 and using the default for all other arguments:


> set.seed(1234)> sampWiener(N = 10, a = 1, v = .3, w = .6,t0 = .2)

The set.seed() function call allows the reader to reproduce the output, which can be seen here:


$q[1] 0.4992622 0.2939385 0.2920679 0.26360800.3015461 0.2564201 0.2723436 0.39614370.7115215 0.4110627$response[1] "upper" "upper" "upper" "upper" "upper""upper" "upper" "upper" "upper" "lower"$callsampWiener(N = 10, a = 1, v = 0.3, w = 0.6,t0 = 0.2)attr(,"class")[1] "Diffusion_samp"

## Results

In the following, we present three different simulation studies and their results. The first simulation study tests log-concavity of the PDF of *A* (see Eq. 5), the second checks whether the four sampling methods produce random samples that are distributed according to the intended first-passage time distributions, and the third evaluates which of the sampling methods is fastest in which condition. In addition, we compared the speed of the WienR package with two other packages that are able to sample random first-passage times from the WDM, namely RWiener (Wabersich & Vandekerckhove, 2014) and rtdists (Singmann et al., 2020). The results of this speed comparison can be found in Appendix C, in which it is shown that our package with the ARS method outperforms the other packages. For all simulation studies, we used the same random seed (2021) in our *R* scripts. All scripts and graphics can be found on https://osf.io/mqtj9/.

Note that in all simulation studies the non-decision time *t*_0_ and its within-person variability $s_{{t}_{0}}$ are not used. This is due to the fact that these parameters do not affect the sampling methods. The non-decision time just adds to the first-passage time and for $s_{t_{0}}>0$ can be sampled separately.

### Log-concavity for ARS

In this first simulation study, we randomly sampled *N* = 10,000 values for each parameter of a WDM with five parameters (excluding non-decision time and its within-person variability). The upper boundary *a* is uniformly sampled from $[0.5,\, 2]$, the mean drift rate $\mu_\nu$ is sampled from a standard normal distribution, the mean starting point $\mu_{w}$ is sampled from a beta distribution with both shape parameters being eight, the within-person variability for the drift rate ${s}_\nu$ is uniformly sampled from $[0,\, 2]$, and the within-person variability of the relative starting point *s*_*w*_ is uniformly sampled from $[0,\, 0.2]$. In addition, the responses are sampled from a Bernoulli distribution with *p* = 0.5.

This resulted in 10,000 random parameter sets. For each of these parameter sets, we generated 500 first-passage times *t*, covering the range from 0.01 to 5 s in steps of 0.01 s. These were transformed to 500 *α* values using Eq. 4. For each *α* we numerically calculated the second derivative of $\log (f_A(\alpha ))$ (see Eq. 5) as follows: The first derivative

7 $$\frac{\partial}{\partial \alpha} \log(f_{A}(\alpha)) = s_{\alpha} \left( \frac{f_{T}^{\prime}(t)}{f_{T}(t)}\times t + 1\right),$$

where $t={v}^{-1}(\alpha )=\exp (s_{\alpha } \alpha + \alpha _{0})$, *f*_*T*_ is the density of the random variable **T** (i.e., the first-passage time variable), and $f_T^{\prime }$ its partial derivative with respect to *t*, can be calculated using the WienR package. Log-concavity would be violated if for any of the 5,000,000 *α* values (10,000 parameter sets times 500 α values per parameter set), the second derivative were positive. Based on the first derivatives, the second derivative can be calculated numerically using the numDeriv package (Gilbert & Varadhan, 2019). Because the numerically computed second derivative is only an approximation of the second derivative itself, it may become positive due to approximation error. In such cases, we calculate it anew with a higher precision for the PDF and its first partial derivative with respect to *t*. Only 246 of the 5,000,000 *α* values had positive numerical second derivatives of the log-density in the first run. Increasing the precision of the numerical calculation and rerunning these 246 cases eliminated these positive numerical second derivatives.^5^

### Accuracy of the sampling methods

A second simulation study checks whether the samples drawn by means of the different methods follow the intended first-passage time distribution. We have four sampling methods (ARS, ITS, RS, P-ARS), used two cut-off values for truncating the distribution from above (infinity and 0.5 s), two variability options for the drift rate (${s}_\nu$ = 0 vs. $s_\nu \sim \mathcal {U}(0,1)$), and two variability options for the relative starting point (*s*_*w*_ = 0 vs. $s_w \sim \mathcal {U}(0, 0.2)$). This leads to eight conditions per sampling method and 32 conditions in total. For each condition, we generated 10,000 random parameter sets using the same fixed random seed as above. The upper boundary *a* was uniformly sampled from $[.6,\, 2]$, the mean drift rate $\mu_\nu$ from a standard normal distribution, and the mean relative starting point $\mu_{w}$ from a beta distribution with both shape parameters being eight. In addition the responses were sampled from a Bernoulli distribution with *p* = 0.5.

For each parameter set, we then sampled 10,000 first-passage time values and checked whether the samples come from the first-passage time distribution with the corresponding parameters using a one-sample Kolmogorov–Smirnov test. This resulted in 10,000 *p* values for each condition. We checked for each condition how many *p* values were below .05 and whether the *p* values come from a standard uniform distribution using again the one-sample Kolmogorov–Smirnov test.

As can be seen in Table 1, the percentage of the 10,000 *p* values smaller than .05 (in parentheses) approaches the expected value of 5% in all 32 conditions. For testing each set of 10,000 *p* values for uniformity, we used the ks.test() function from the stats package (R. Core Team, 2020). Three of the *p* value samples differ significantly from uniformity, one for the ITS, and two for the RS method. The probability of observing three or more significant outcomes of the Kolmogorov–Smirnov test across the 32 conditions is .21.^6^ Looking at the histograms of all three corresponding *p* value samples, there is no clear pattern indicating a deviation from uniformity (see Figs. 3, 4, and 5 in Appendix B). Altogether, the results speak in favor for a correct implementation of the four sampling methods.

**Table 1:** The *p* values from testing for uniformity and in parentheses the proportion of significant tests when testing random first-passage time samples

Tr.	${s}_\nu$ = 0		${s}_\nu$ > 0	
	*s*_*w*_ = 0	*s*_*w*_ > 0	*s*_*w*_ = 0	*s*_*w*_ > 0
ARS				
0.5	.946 (.049)	.702 (.046)	.314 (.045)	.915 (.047)
Inf	.201 (.045)	.499 (.046)	.554 (.046)	.133 (.048)
ITS				
0.5	.401 (.047)	.134 (.051)	.807 (.048)	.950 (.051)
Inf	.471 (.047)	.117 (.049)	.045 (.049)	.627 (.048)
RS				
0.5	.817 (.050)	.691 (.048)	.110 (.051)	.015 (.047)
Inf	.056 (.050)	.111 (.051)	.042 (.044)	.935 (.051)
P-ARS				
0.5	.730 (.047)	.331 (.049)	.315 (.052)	.311 (.050)
Inf	.119 (.043)	.245 (.052)	.757 (.051)	.059 (.045)

*Note.* ARS, ITS, RS, and P-ARS denote the four different sampling methods, adaptive rejection sampling, inverse transform sampling, rejection sampling, and pseude-adaptive rejection sampling, respectively. “Tr.” denotes truncation from above when using the different sampling methods; Inf = infinity (i.e., no truncation) and 0.5 means no sample will be larger than 0.5 s. ${s}_\nu$ and $s_{w}=0\ (> 0)$ means that the within-person variability for the drift rate and relative starting point, respectively, is zero (larger than zero). The observed proportion of significant (*p* < .05) one-sample Kolmogorov–Smirnov tests (in parentheses) is computed from testing 10,000 random first-passage time samples (in the just mentioned conditions) for each of 10,000 random parameter sets. The resulting 10,000 *p* values (from all parameter sets) were then tested for uniformity using the one-sample Kolmogorov–Smirnov tests, resulting in 32 *p* values over all conditions.

### Speed of the samplers

The speed of the four sampling methods was measured by the package bench (Hester, 2020). We compared the speed of the sampling methods for two truncation options (infinity vs. 0.5 s), two boundary options (sampling from both bounds vs. from only one random boundary), two variability options for the drift rate (${s}_\nu$ = 0 vs. $s_\nu \sim \mathcal {U}(0,2)$), and two variability options for the relative starting point (*s*_*w*_ = 0 vs. $s_w \sim \mathcal {U}(0, 0.2)$), leading to 16 conditions. In addition, we varied the number of values sampled per simulation run in seven levels: Sample sizes were *N* = 10^*i*^, $i \in \{0, ..., 6\}$. To get a valid estimate of the speed, the median of 100 iterations was taken for each condition. The four core parameters—upper boundary *a*, drift rate $\nu$, relative starting point *w*, and response—were sampled like in Section “Accuracy of the sampling methods”.

**Fig. 2:**
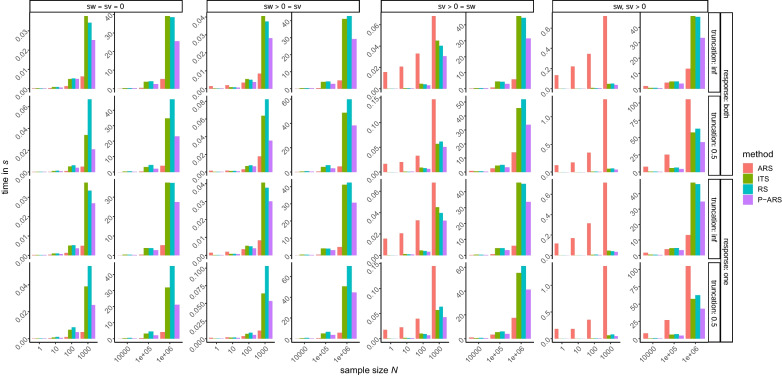
Speed comparison for all conditions (response, truncation, and within-person variability). *Note.* “method” stands for the four sampling methods, namely ARS = adaptive rejection sampling, ITS = inverse transform sampling, RS = rejection sampling, and P-ARS = pseudo-adaptive rejection sampling. The terms sw and sv stand for the within-person variability for the relative starting point and the drift rate, respectively; a value of 0 means no variability and a value larger than 0 means there is variability. “response: both” means that the first-passage times are sampled from both boundaries and “response: one” means that they are sampled from one boundary. “truncation: inf” means no truncation and “truncation: 0.5” means a truncation from above at 0.5 s. A sample size *N* of 1e+05 means 100,000 and 1e+06 means 1,000,000

**Fig. 3:**
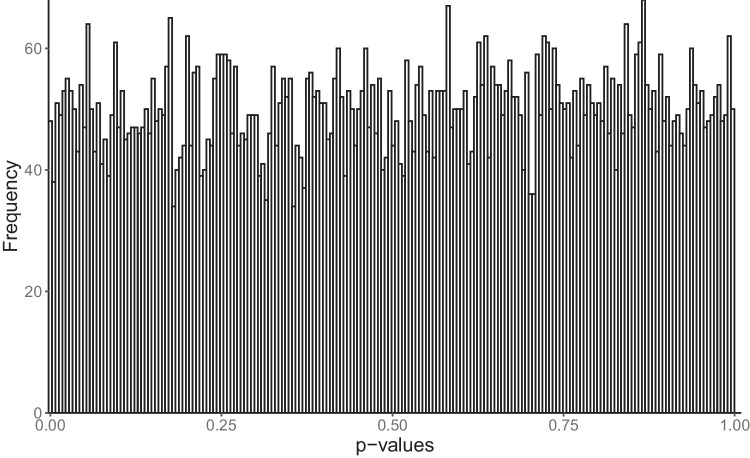
Histogram of *p* values for the ITS method without truncation and within-person variability for $\nu$

**Fig. 4:**
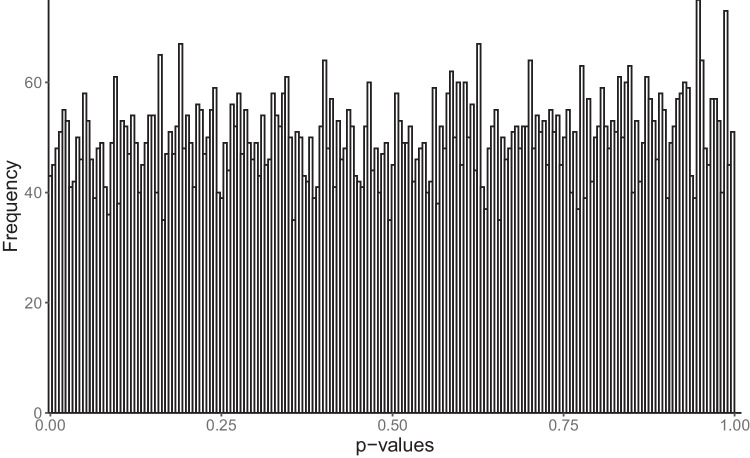
Histogram of *p* values for the RS method with truncation and both within-person variabilities

**Fig. 5:**
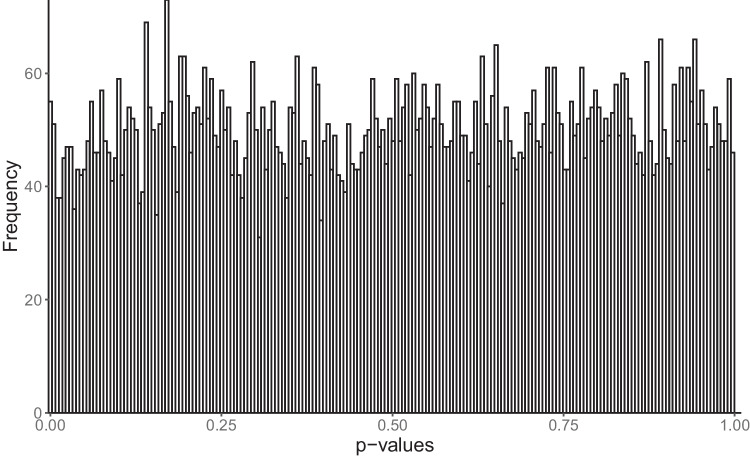
Histogram of *p* values for the RS method without truncation and within-person variability for $\nu$

The first row of Fig. 2 depicts the speed comparisons between the four sampling methods (ARS, ITS, RS, and P-ARS) for the no-truncation condition with sampling from both boundaries for the four variability conditions, in order, $s_\nu= s_{w}=0,\, s_{w} > 0=s_\nu,\, s_\nu > 0= s_{w}, \, \mathrm{and}\; s_\nu,\, s_{w}> 0$. These four figures show the same pattern for all the sampling methods as the previous four figures: With more within-person variability ARS requires more time, but as *N* increases, the sampling time increases less steeply than for the other methods. P-ARS is comparable to ITS and RS, but always at least slightly faster for *N* > 10.

The second row of Fig. 2 depicts the speed comparisons between the four sampling methods (ARS, ITS, RS, and P-ARS) for the truncation condition (truncation from above at 0.5 s) with sampling from both boundaries for the same four variability conditions. The ARS method is fastest for *N* > 1 with no within-person variability, for *N* > 100 with within-person variability in *w* only, for *N* > 100,000 with within-person variability in $\nu$ only, but never with both variabilities. The P-ARS method performs best or second best for *N* > 100, depending on the performance of the ARS method.

The third row of Fig. 2 depicts the speed comparisons between the four sampling methods (ARS, ITS, RS, and P-ARS) for the condition without truncation and with sampling from only one random boundary for the four variability conditions. As can be seen, the ARS method has an advantage when *N* increases. It is superior with *N* > 10 when using no within-person variability, with *N* > 100 when using within-person variability in *w *only, with *N* > 1,000 when using within-person variability in $\nu$ only, and with *N* > 1,000,000 when using within-person variability in *w* and $\nu$. For *N* > 10, the P-ARS method is always second fastest or fastest, depending on the performance of the ARS method. Third and fourth place are taken by the RS and ITS methods, for which the pattern is not always as clear as for ARS and P-ARS.

The fourth row of Fig. 2 depicts the speed comparisons between the four sampling methods (ARS, ITS, RS, and P-ARS) for the truncation condition (truncation from above at 0.5 s) with sampling only from one random boundary for the four variability conditions. Here the general pattern is similar, but the ARS performs worse than without truncation; the ARS method is superior with *N* > 1 when using no within-person variability, with *N* > 100,000 when using within-person variability either in *w* or $\nu$, and never when using within-person variability in *w* and $\nu$, at least not with *N* up to 1,000,000. The P-ARS method is mostly second fastest or fastest, depending on ARS and RS. Especially for *N* < 100 the RS method is most often the fastest. When using within-person variability with large sample sizes (about *N* = 1,000,000) the ITS and P-ARS perform comparably; sometimes ITS is better and sometimes P-ARS.

## Discussion

We proposed new methods for sampling from first-passage time distributions arising under the WDM based on adaptive rejection sampling and evaluated these methods as well as two alternatives for sampling from these distributions. The present comparison was restricted to sampling methods that intend to sample from the exact distributions rather than from approximations thereof.

In simulation studies, we checked whether the methods do indeed sample from the target distributions and results suggest that they do. Further simulation studies compared the speed of the different methods in generating random values. Here, it turned out that overall, the new ARS method has the largest speed advantage when within-person variability of drift rate and starting point is set to zero and more than one sample is to be drawn with the same parameter values. For the cases with within-person variabilities, the ARS method is initially the slowest, but in sampling without truncation, overtakes the other sampling methods when *N* is larger than 10,000. This suggests that the adaptive envelope and squeezing functions constructed in the course of ARS sampling approximate the true PDF increasingly more closely, as sampling proceeds, so that costly evaluations of the first-passage time density become increasingly rare. The P-ARS method can be used whenever the ARS method is slow, namely when using within-person variabilities, especially in connection with truncation. Given truncation and within-person variabilities, the RS and ITS methods are comparable to P-ARS when *N* < 1,000.

The absolute size of the speed differences in Fig. 2 is small and will often be negligible. Having a fast sampling method is, however, helpful whenever applications rely on extensive simulations. For example, we developed the new sampling algorithms in the context of constructing an MCMC algorithm for fitting a complex, non-standard WDM at the core of which is the WDM without within-person variability (such variabilities are modeled separately at a higher hierarchical level in Bayesian modeling using the WDM; Vandekerckhove et al., 2011). In this algorithm, a truncated first-passage time distribution is involved in a rejection-sampling step with relatively low acceptance rate. Each such step typically involves the sampling of thousands of random first-passage times, and the step is iterated countless times as the algorithm converges.

Other examples involve cognitive models in which the diffusion model is a building block, but in which additional processes are involved with associated response time distributions. For example, in the dual-stage two-phase model of response-conflict tasks (Hübner et al., 2010), three diffusion processes interact to produce observed responses and response times. The first two of them run in parallel. If the first diffusion process reaches a boundary first, the response and response time is based on that process. Otherwise, if the second diffusion process terminates first, a third diffusion process is initiated after completion of the second diffusion process. In that case, the observed response is determined by the third process with overall response time given by the sum of the second and third diffusion process’ first-passage times. The likelihood function of this and similar models is very difficult to compute, but it is easy to sample from it by generating responses and response times for the three involved diffusion process. This makes it a prime candidate for likelihood-free estimation methods such as the probability density approximation (Turner & Sederberg, 2014) that rely on the ability to generate large samples of simulated data for each of many parameter values.

As a final example, consider posterior predictive model checks in Bayesian posterior inference (Gelman et al., 2004) in which aspects of the observed data and simulated data are contrasted with the values expected on the basis of the parameters from each set of parameter values in the usually large sample of parameter sets drawn from the posterior distribution of parameters. For example, such model checks often assess whether the posterior distribution of parameter values adequately accounts for the observed means and variances of the response time distributions (see Klauer & Kellen, 2018, for examples of model checks of this kind). This involves computing the expected means and variances of first-passage times for many parameter values. These are again very difficult to compute for diffusion models involving within-person variabilities, but it is easy to estimate them with adequate precision if large samples of first-passage times can be efficiently generated. In all of these and similar contexts, it is essential to have a highly efficient method for generating random first-passage times.

The newly implemented *R* function sampWiener() enriches the WienR package by providing multiple sampling methods to sample from the (truncated) first-passage time distribution of a WDM with 3–7 parameters. This enables the user to choose the sampling method that is best suited to his or her application according to the guidelines derived above.

## Figures and Tables

**Fig. 6:**
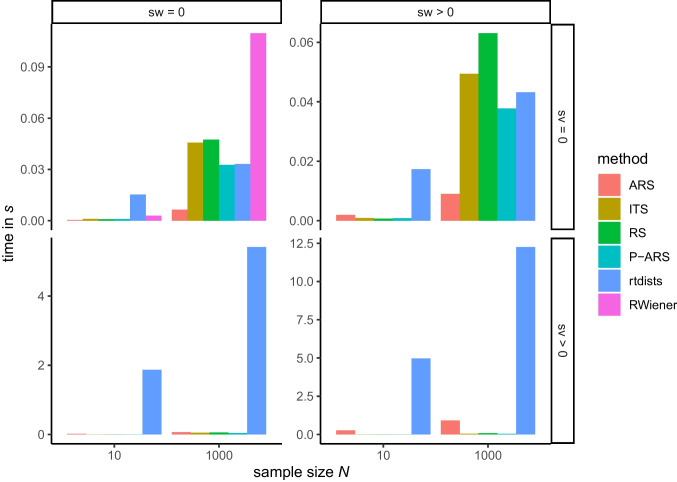
Speed comparison of the four WienR sampling methods with the RWiener-package and the rtdists-package. *Note.* sw and sv stand for the within-person variability in the relative starting point and the drift rate, respectively. For example, sv = 0 means no variability in the drift rate and sv> 0 means with variability for the drift rate. “methods” are the four sampling methods, namely ARS = adaptive rejection sampling, ITS = inverse transform sampling, RS = rejection sampling, and P-ARS = pseudo-adaptive rejection sampling. “rtdists” and “RWiener” are two other packages with which it is possible to sample first-passage times. Note, however, that “RWiener” is only able to sample first-passage times without using within-person variabilities and “rtdists” is only able to use a much lower precision (roughly about 1e-4) than the other methods/packages (here 1e-10); higher precision values would lead to much higher sampling times

## Appendix A: Log-concavity of the inverse Gauss PDF with change-of-variables

Let **T** be inverse Gauss distributed with *μ* > 0 being the mean and *λ* > 0 the shape parameter. The PDF of **T** is given by

8 $$f_{T}(t; \mu,\lambda) = \sqrt{\frac{\lambda}{2\pi t^{3}}} \times \exp\left( -\frac{\lambda (t-\mu)^{2}}{2\mu^{2}t}\right).$$

Using the formula for change-of-variables with $\mathbf {A}=(\log (\mathbf {T})-\alpha _{0})/s_{\alpha }$ (see also Eq. 4) gives

9 $$f_{A}(\alpha; \mu, \lambda) = s_{\alpha} \exp\left( s_{\alpha} \alpha + \alpha_{0}\right) \times f_{T}(\exp\left( s_{\alpha} \alpha + \alpha_{0}\right); \mu, \lambda).$$

Taking the logarithm of Eq. 9 and using that $t(\alpha ) = \exp (s_{\alpha }\alpha +\alpha _{0})$ gives

10 $$\begin{aligned} \begin{aligned}\begin{array}{@{}rcl@{}} \log(f_{A}(\alpha; \mu, \lambda)) &=& \log(s_{\alpha}) + s_{\alpha}\alpha + \alpha_{0} + \frac{1}{2} \log(\lambda) - \frac{1}{2} \log(2\pi t(\alpha)^{3}) - \frac{\lambda(t(\alpha) - \mu)^{2}}{2\mu^{2}t(\alpha)}\\ &=& \log(s_{\alpha}) - \frac{1}{2} s_{\alpha}\alpha - \frac{1}{2} \alpha_{0} + \frac{1}{2} \log(\lambda) - \frac{1}{2} \log(2\pi) - \frac{\lambda(t(\alpha) - \mu)^{2}}{2\mu^{2}t(\alpha)}. \end{array}\end{aligned} \end{aligned}$$

Taking the first derivative with respect to *α* gives us

11 $$\frac{\partial \log(f_{A}(\alpha; \mu, \lambda))}{\partial \alpha} = - \frac{1}{2} s_{\alpha} - \frac{\lambda s_{\alpha} (\exp(2s_{\alpha}\alpha+2\alpha_{0})-\mu^{2})}{2\mu^{2}\exp(s_{\alpha}\alpha+\alpha_{0})}$$

and then taking the second derivative gives us

12 $$\frac{\partial^{2} \log(f_{A}(\alpha; \mu, \lambda))}{\partial \alpha^{2}} = - \frac{\lambda s_{\alpha}^{2} (\exp(2s_{\alpha}\alpha+2\alpha_{0})+\mu^{2})}{2\mu^{2}\exp(s_{\alpha}\alpha+\alpha_{0})},$$

which is clearly negative. Therefore the PDF of an inverse Gauss with change-of-variables ${v}(\mathbf{T})$ is log-concave.

## Appendix C: Speed comparison with other R packages

Figure 6 shows the median sampling time (of 100 repetitions for a sample size of 10 and 1000 each) needed for the four sampling methods of WienR, the rtdists-package, and the RWiener-package (only for the case with no within-person variability) in the four variability conditions; no within-person variability (i.e., sw= 0 and sv= 0), within-person variability only for the relative starting point (i.e., sw> 0 and sv= 0), within-person variability only for the drift rate (i.e., sw= 0 and sv> 0), and within-person variability for both parameters (i.e., sw> 0 and sv> 0). The samples are drawn from both boundaries and without truncation.

Note that the precision for all methods are set to 1e-10 (which is fixed for RWiener) except for rtdists. For the package rtdists, we set the precision argument to 4 which roughly corresponds to a precision of 1e-4. A higher precision would take too long, especially for the cases with within-person variability of the drift rate, as can be seen in the bottom row of Fig. 6 where the differences to the methods of WienR are already quite large.

For cases with no variability in the drift rate and a sample size of 1000 the rtdists-package is about as fast as most of the other WienR-methods but still three to four times slower than ARS. For cases where the sample size is 10, it clearly performs worst. The package RWiener performs worst for a sample size of 1000 and second to last for a sample size of 10. Overall, the ARS and P-ARS methods outperform rtdists as well as RWiener in all conditions.

## Footnotes

1 These parameters are chosen conditional on *a*, $\nu$, *w*, and the response so that $\exp (\alpha _{0})$ is the expected mean of **T** and $\exp (s_{\alpha })$ close to its standard deviation.

2 For cases where within-person variability is used for $\nu$, *w*, or *t*_0_ the absorption probability is the CDF at $t=\infty$. In a similar vein, for cases where the samples are to be drawn with truncation at *T*_trunc_ the CDF for the upper (CDF_upper_) and the lower bound (CDF_lower_) need to be calculated at $t=T_{\mathrm{trunc}}$ and the absorption probability for the upper boundary will be $\mathrm{CDF}_{\mathrm{upper}}/(\mathrm{CDF}_{\mathrm{upper}}+ \mathrm{CDF}_{\mathrm{lower}})$.

3 When the absorption probability at a given boundary (see Eq. 6) becomes very small, computation of the conditional PDF and CDF given absorption at that boundary becomes numerically unstable. However, the current algorithm mostly works with the joint PDF and CDF for absorption boundary and response latency (e.g., with $\mathrm{P}(T < t\, \&\, \mathrm{Absorption} = \mathrm{upper})$ instead of $\mathrm{P}(T < t \mid \mathrm{Absorption} = \mathrm{upper}))$, which avoids the issue. To increase efficiency the four sampling methods are implemented in *C++* and can be called through *R* like the other functions of WienR (see the package source code, e.g., at CRAN).

4 Since names like rwiener (Wabersich & Vandekerckhove, 2014) or rdiffusion (Singmann et al., 2020) are already used by other packages we decided to use another notation.

5 The same pattern of results was found when not using both within-person variabilities (i.e., setting either $s_\nu=0, s_{w}=0, \mathrm{or}\, s_\nu=s_{w}=0$); that is, there were no positive second derivatives for any *α* value.

6 We repeated the whole analysis with two different random seeds (2020 and 2022). For both seeds, the number of observed significant Kolmogorov–Smirnov tests across the 32 conditions was not significant. The significant tests scatter randomly over the sampling methods and the conditions.
